# Incorporation of Ebola glycoprotein into HIV particles facilitates dendritic cell and macrophage targeting and enhances HIV-specific immune responses

**DOI:** 10.1371/journal.pone.0216949

**Published:** 2019-05-17

**Authors:** Zhujun Ao, Lijun Wang, Emelissa J. Mendoza, Keding Cheng, Wenjun Zhu, Eric A. Cohen, Keith Fowke, Xiangguo Qiu, Gary Kobinger, Xiaojian Yao

**Affiliations:** 1 Laboratory of Molecular Human Retrovirology, Max Rady College of Medicine, Rady Faculty of Health Sciences, University of Manitoba, Winnipeg, Manitoba, Canada; 2 Department of Medical Microbiology, Max Rady College of Medicine, Rady Faculty of Health Sciences, University of Manitoba, Winnipeg, Manitoba, Canada; 3 Department of Histology and Embryology, Zunyi Medical College, Zunyi, Guizhou, China; 4 Zoonotic Diseases and Special Pathogens, National Microbiology Laboratory, Public Health Agency of Canada, Winnipeg, Manitoba, Canada; 5 Institut de Recherches Cliniques de Montréal, Montreal, Quebec, Canada; Département de Microbiologie, Infectiologie et Immunologie, Université de Montréal, Montreal, Quebec, Canada; 6 Centre de Recherche en Infectiologie de l’Université Laval/Centre Hospitalier de l’Université Laval (CHUL), Québec, Quebec, Canada; George Mason University, UNITED STATES

## Abstract

The development of an effective vaccine against HIV infection remains a global priority. Dendritic cell (DC)-based HIV immunotherapeutic vaccine is a promising approach which aims at optimizing the HIV-specific immune response using primed DCs to promote and enhance both the cellular and humoral arms of immunity. Since the Ebola virus envelope glycoprotein (EboGP) has strong DC-targeting ability, we investigated whether EboGP is able to direct HIV particles towards DCs efficiently and promote potent HIV-specific immune responses. Our results indicate that the incorporation of EboGP into non-replicating virus-like particles (VLPs) enhances their ability to target human monocyte-derived dendritic cells (MDDCs) and monocyte-derived macrophages (MDMs). Also, a mucin-like domain deleted EboGP (EboGPΔM) can further enhanced the MDDCs and MDMs-targeting ability. Furthermore, we investigated the effect of EboGP on HIV immunogenicity in mice, and the results revealed a significantly stronger HIV-specific humoral immune response when immunized with EboGP-pseudotyped HIV VLPs compared with those immunized with HIV VLPs. Splenocytes harvested from mice immunized with EboGP-pseudotyped HIV VLPs secreted increased levels of macrophage inflammatory proteins-1α (MIP-1α) and IL-4 upon stimulation with HIV Env and/or Gag peptides compared with those harvested from mice immunized with HIV VLPs. Collectively, this study provides evidence for the first time that the incorporation of EboGP in HIV VLPs can facilitate DC and macrophage targeting and induce more potent immune responses against HIV.

## Introduction

Acquired immunodeficiency syndrome (AIDS) is a slow degenerative disease of the immune and nervous systems resulting from HIV infection. The pathogenesis of HIV-1 infection is linked closely to the replication of the virus in CD4^+^ helper T cells followed by the massive loss of CD4^+^ T cells *in vivo* [1,2]. Although the development of highly active antiretroviral therapy (HAART) has significantly improved the prognosis of HIV-infected individuals, it is unable to permanently eliminate HIV infection and it is ineffective against drug-resistant HIV variants [3]. Providing a long-term intervention with HAART is limited in its ability to control the global epidemic due to cost and availability issues in developing countries where a substantial portion of the HIV positive population exists. Thus, the development of efficient protective vaccines to prevent HIV infection has become a major priority. The immunogenicity and protective efficacy of several vaccine strategies against HIV infection have been evaluated *in vivo* (reviewed in [4]). Among all the vaccines tested in non-human primate (NHP) models with simian immunodeficiency virus (SIV), live attenuated SIVs remain the most efficacious vaccines in protecting against wild-type SIV [5]. However, a live attenuated HIV vaccine for humans is unfeasible due to considerable safety issues. Therefore, much effort has been made to develop other types of vaccines, including inactivated HIV virions and virus-like particles (VLPs) [6–9].

Dendritic cells (DCs) are a crucial link between the innate and adaptive immunity, presenting antigens upon infection and activating naïve lymphocytes for induction of cytolytic and memory responses [10,11]. In addition, DCs release specific cytokines which drive the Th1 and/or Th2 arm(s) of T cell response against pathogens [12]. Given the importance of DCs in the regulation of the immune response and their ability to act as immunologic activators and natural adjuvants [13], DC-based immunotherapeutic vaccines have become a growing field in HIV therapeutic development [14–17]. In these approaches, autologous DCs are stimulated with HIV immunogens, including inactivated HIV-1 particles, HIV-1 peptides, and autologous HIV-infected apoptotic cells, and allowed to mature with a combination of various cytokines *in vitro*. The stimulated DCs are subsequently injected into the corresponding patient to prime potent HIV-specific cytotoxic T lymphocyte (CTL) responses, aiming to clear the HIV latent reservoir (reviewed in [18]). However, the biosafety and quality requirements make DC-based immunotherapy a costly method, limiting its extensive clinical application, especially in low-income countries. A novel HIV vaccine strategy targeting DCs could provide a more practical and cost-effective alternative.

In contrast to HIV that targets CD4^+^ T cells, Ebola virus (EBOV) preferentially target the antigen-presenting cells (APCs), including DCs and monocyte-derived macrophages (MDMs) via the EBOV glycoprotein (EboGP) [19,20]. Also, Ebola glycoprotein (EboGP) has been shown to stimulate human DCs to enhance innate and adaptive immune responses through NF-κB and MAPK signaling pathways [21]. Impressively, recent studies (including a clinic trial report) showed that a Vesicular Stomatitis Virus (VSV)-vectored vaccine expressing EboGP demonstrated promise in terms of safety and efficacy [22,23]. Thus, these studies suggest the possibility that EboGP may have potential to direct an HIV vaccine towards DCs while acting as a strong adjuvant to drive effective immune responses. In addition, it has been reported that there is structural similarity between EboGP and retroviral envelope proteins [24] and the EboGP-pseudotyped lentiviral vector has been generated for various research [25,26].

We therefore hypothesized that pseudotyping a non-replicating HIV virus-like particle (VLP) vaccine platform with EboGP could improve the immunogenicity of HIV by directing the antigens towards DCs and macrophages, while enhancing host immune responses against HIV. In this study, we produced HIV VLPs bearing HIV gp or/and EboGP and demonstrated that in the presence of EboGP, HIV VLPs are able to target human DCs and macrophages more efficiently than HIV VLPs alone. Importantly, we demonstrated that mice immunized with the EboGP/HIVgp-pseudotyped HIV VLPs develop significantly stronger HIV Env- and Gag-specific immune responses. Thus, incorporation of EboGP in HIV VLPs can enhance the immunogenicity of HIV and present as potent DC-targeted anti-HIV vaccine approach.

## Results

### Expression of EboGP in HIV virus like particles (VLPs) enhances VLP’s entry into human DCs and macrophages, but not in CD4^+^ T cells *in vitro*

**Fig 1:**
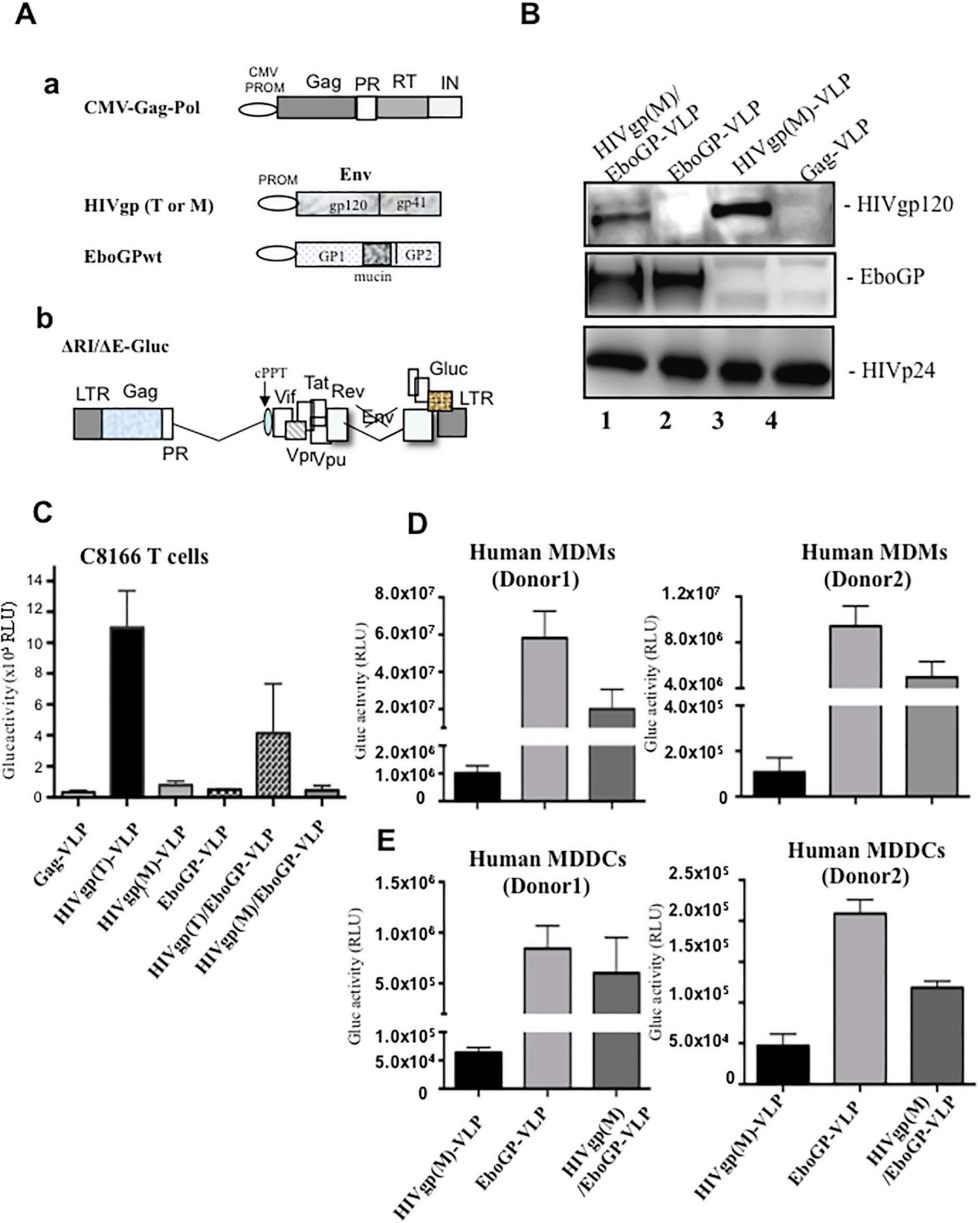
The expression of EboGP in HIV VLPs facilitates virus entry in MDMs and MDDCs, but not in CD4^+^ T cells. A) Schematic structures of various plasmids. a, CMV-Gag-Pol, HIV gp(T/M), EboGP plasmids. CMV PROM: Cytomegalovirus promoter. b, HIV-1 RT, IN and Env deletion provirus ΔRI/ΔE/Gluc. RT: Reverse transcriptase; IN: Integrase; Env: Envelope; Gluc: Gaussia luciferase. B) The presence of EboGP or HIV gp in HIV VLPs. 293T cells were transfected by HIV-1ΔRI/ΔE/Gluc+, CMV-Gag-Pol and HIV gp or EbovGPwt/ΔM. Supernatant containing VLPs were collected, purified, lysed and analyzed by WB with anti-HIVgp, anti-EboGP antibody or anti-HIVp24 antibody. **C-E)** CD4^+^ C8166 T cells, human MDMs and human MDDCs were infected by various HIVgp or EboGP pseudotyped HIV VLPs. After 3 days of infection, the supernatants were collected and subjected to Gluc activity assay. Error bars represent variation between duplicate samples and the data is representative of results obtained in three independent experiments.

Previous studies have demonstrated that EboGP is efficiently incorporated into HIV virions and mediate the entry and fusion of virus into MDMs and MDDCs [25,27,28]. To test whether the incorporation of EboGP into HIV VLPs could alter HIV cell tropism and enhance VLPs targeting human MDMs/MDDCs, we co-transfected a HIV Gag/Pol expression plasmid (CMV-Gag/Pol) [29] and a plasmids encoding EboGP and/or HIVgp (M tropic or M) (Fig 1A,a) into HEK 293T cells. To monitor the DC/macrophage targeting efficiency of VLPs, we also added a HIV vector (ΔRI/ΔE/Gluc) which contain a secreted Gaussia luciferase (Glu) gene in the position of *nef* (Fig 1A,b) in the transfection mix and generated the VLPs containing GLuc gene (ΔRI/ΔE/Gluc). After 48 hrs of transfection, the pseudotyped VLPs were ultracentrifugated, lysed in Laemmli sample buffer and analyzed by western blot (WB), as described in Materials and Methods. As expected, EboGP was detected in HIVgp(M)/EboGP-VLPs and EboGP-VLPs (Fig 1B, lanes 1 and 2), while HIV gp120 was detected in HIVgp(M)/EboGP-VLP and HIVgp(M)-VLPs (Fig 1B, lanes 1 and 3). It is worth noting that the incorporation level of HIVgp(M) was slightly reduced in VLPs expressing both HIVgp(M) and EboGP (Fig 1B, lane 1).

To evaluate their ability to target various cells, equivalent amounts of HIVgp (T or M) or/and EboGP pseudotyped ΔRI/ΔE/Gluc VLPs (normalized with HIV p24 levels) were used to infect CD4+ C8166 T cells, human MDMs, and MDDCs. The entry of the pseudotyped virus can be monitored by detecting the Gluc activity in the culture medium since the Gluc protein will be expressed and released when the virus subsequently replicate. The supernatants were collected and the Glu activities were monitored after 3 days for C8166 cells and 8 days for human MDMs and MDDCs. As expected, the results showed that HIVgp(T)-VLPs can efficiently infect and produced high Gluc activity in CD4+ C8166 T cells, while HIVgp(M)-VLPs and HIVgp(M)/EboGP-VLPs infection did not produce significant levels of Gluc activity as compared to negative control (Gag-VLP) (Fig 1C). In contrast, in MDMs and MDDCs from two health donors, HIVgp(M)/EboGP-VLPs and EboGP-VLPs mediated very efficient virus entry and replication since significantly higher Gluc activities were detected, as compared to HIVgp(M)-VLPs (Fig 1D and 1E). However, the infectivity of HIVgp(M)/EboGP-VLPs was lower than that of EboGP-VLPs even though there was a similar level of EboGP in those VLPs (Fig 1B, 1D and 1E). The mechanism underlying the various infectivity levels is not clear. It could be possible that the presence of HIVgp(M) on the HIVgp(M)/EboGP-VLPs may somehow interfere with the function of EboGP, such as binding to its receptors and subsequently influence VLPs entry into the cells. Overall, these results indicate when pseudotyped with EboGP, HIV VLPs can more efficient target and enter into human MDMs and MDDCs.

### HIV VLPs pseudotyped with EboGPΔM display more efficiency for entering MDMs and MDDCs

**Fig 2:**
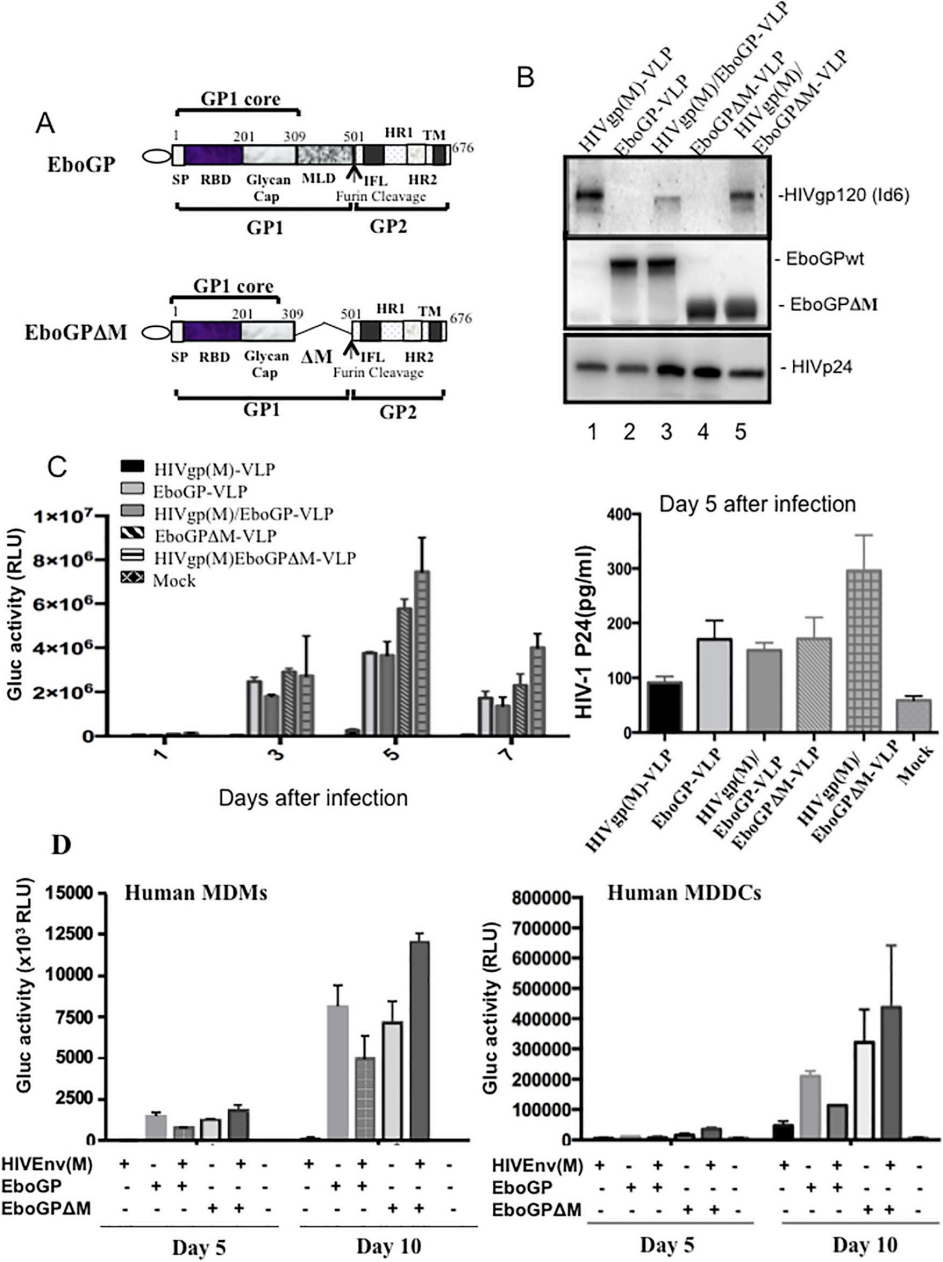
The EboGPΔM incorporation into HIV VLPs mediates more efficient virus entry in MDMs and MDDCs than EboGP. A) Schematic diagram of the peptide sequence for EboGPwt and EboGPΔM, which contained a deletion of the MLD region. SP: signal peptide; RBD: receptor binding domain; MLD: mucin-like domain; IFL: internal fusion loop; HR1, HR2: heptadrepeat1/2; TM: transmembrane domain. **B)** Analysis of the viral compositions of various VLPs produced from 293T cells co-transfected with HIV Env(M) and/or EboGP/EboGPΔM expressing plasmids, and HIV CMVin-Gag/Pol and HxΔRI/ΔE/Gluc. Viral compositions were analyzed by WB with anti-HIVgp, anti-EboGP antibody or anti-HIVp24 antibody **C)** THP1 cells were infected by various HIVgp and/or EboGPwt/ΔM pseudotyped HIV VLPs. At 1, 3, 5, and 7 days post-infection, the supernatants were collected and subjected to Gluc activity and p24 ELISA assay. D) Human MDMs (left panel) or human MDDCs (right panel) were infected by various HIVgp and/or EboGPwt/ΔM pseudotyped HIV VLPs and Gluc activity was monitored at 5 and 10 days post-infection. Error bars represent variation between duplicate samples, and the data is representative of results obtained in two independent experiments.

In the EboGP, there is a mucin-like domain (MLD) which is a highly glycosylated region spanning residues 309 to 501 (Fig 2A), which has multiple functions during EBOV infection *in vivo* but is dispensable for EBOV infection *in vitro* [30]. Removal of this MLD region was previously shown to enhance EboGP-mediated lentiviral vector entry in epithelial cells [25]. Therefore, we constructed an MLD-deleted EboGP (EboGPΔM) expressing plasmid (Fig 2A) and generated the EboGPΔM pseudotyped HIV VLPs (EboGPΔM-VLPs), as described in Materials and Methods. The WB analysis revealed that EboGPΔM protein was efficiently incorporated in the VLPs and the protein migrated faster than EboGP, which had molecular weight of about 75 kDa (Fig 2B, lanes 4 and 5). Interestingly, the analysis revealed that more HIV glycoproteins were incorporated into HIVgp/EboGPΔM-VLPs than that of HIVgp/EboGP-VLPs (Fig 2B, compare lane 5 to lane 3).

To compare the abilities of EboGP and EboGPΔM to promote HIV VLP entry into monocytes, MDMs and MDDCs cells, we first infected THP-1 cells, a human monocytic cell line derived from an acute monocytic leukemia patient [31]. At different time intervals, supernatants of the VLP-treated cell cultures were collected and the Gluc activities and HIVp24 levels were measured. Results showed that the EboGPΔM-VLPs exhibited slightly higher cell entry into THP-1 cells compared with EboGP-VLPs. Interestingly, HIVgp(M)/EboGPΔM-VLPs mediated the highest entry efficiency than others (Fig 2C). To further confirm above observations in primary cells, the human MDMs and MDDCs were isolated and infected with equal amounts of different VLPs, and at days 5 and 10 of post infection, Gluc activities in the supernatants were measured. The results clearly indicated that both EboGP and EboGPΔM were able to mediate more efficient entry of HIV VLPs into human MDMs and MDDCs. Consistent with the findings in the THP-1 cell line, EboGPΔM-pseudotyped HIV VLPs still exhibited stronger DC/MDM-targeting ability than EboGP-pseudotyped HIV VLPs (Fig 2D). All together, the data provided evidence that the EboGPΔM-VLPs, especially the EboGPΔM/ HIVgp(M)-VLPs mediated the stronger entry efficiency than others (Fig 2D).

### EboGP pseudotyped HIV VLPs induced significantly higher specific anti-HIV antibodies than native HIV VLPs in mice

**Fig 3:**
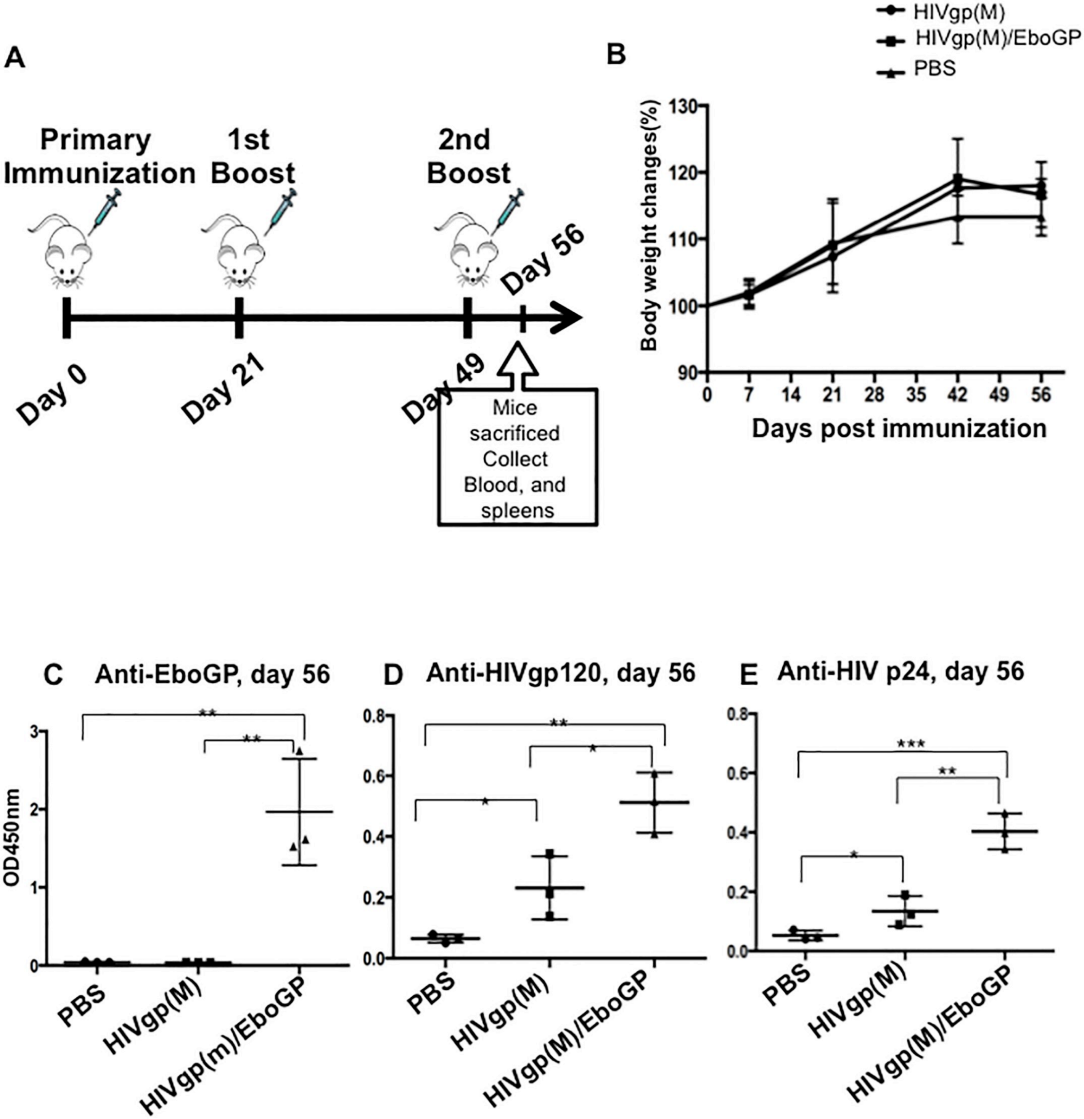
Significantly higher levels of anti-HIV antibodies induced by EboGP-pseudotyped HIV VLPs compared with native HIV VLPs in C57BL/6 mice. A) Schematic of the HIV Env or EboGP-pseudotyped HIV VLP immunization protocol used in this study. The C57BL/6 mice were injected subcutaneously with 100 ng of HIVgp-VLPs, or EboGP/HIVgp-VLPs, as indicated. At 21 and 49 days post-immunization, mice were boosted with same amounts of VLPs, and sera and spleens were collected at 56 days of post-immunization. B) Mice body weights were monitored weekly in which 100% body weight was set at day 0. The levels of anti-EboGP **(C)**, anti-HIVgp120 **(D)**, anti-HIVp24 **(E)** antibodies in the sera of immunized C57BL/6 mice were detected by corresponding ELISAs. The value is the individual amount for each mouse of each group. Statistical significance was determined using unpaired t—test, and significant *p* values are represented with asterisks, *≤0.05, **≤0.01, ***≤0.001.

Since EboGP pseudotyping HIV VLPs significantly enhanced targeting towards MDDCs/MDMs, we next investigated whether the incorporation of EboGP into HIV VLPs could increase the immunogenicity of HIV VLPs *in vivo*. As depicted in Fig 3A, we first immunized C57BL/6 mice with 100 ng of HIVgp(M)-VLPs or HIVgp(M)/EboGP-VLPs on days 0, 21 and 49. Meanwhile, the body weight of all groups of immunized mice were monitored. The body weight of HIVgp(M)-VLPs and HIVgp(M)/EboGP-VLPs groups display a slight increase but there was no statistically significance between groups (Fig 3B). All groups of immunized mice remained healthy. At day 56 of post-immunization, the mice sera were collected, as described in Materials and Methods, and the anti-HIVgp120, -HIVp24 and -EboGP-specific humoral response were measured by corresponding ELISA. As shown in Fig 3, HIV-specific humoral immune responses against HIV Env and Gag were detected in mice injected with HIVgp(M)-VLPs and HIVgp(M)/EboGP-VLPs (D and E), while anti-EboGP humoral immune responses were only detected in the mice injected with HIVgp(M)/EboGP-VLPs (Fig 3C). Interestingly, results showed that the gp120 and p24-specific antibody titers for mice immunized with HIVgp(M)/EboGP-VLPs were significantly higher than that found with HIVgp(M)-VLPs (Fig 3D and 3E).

**Fig 4:**
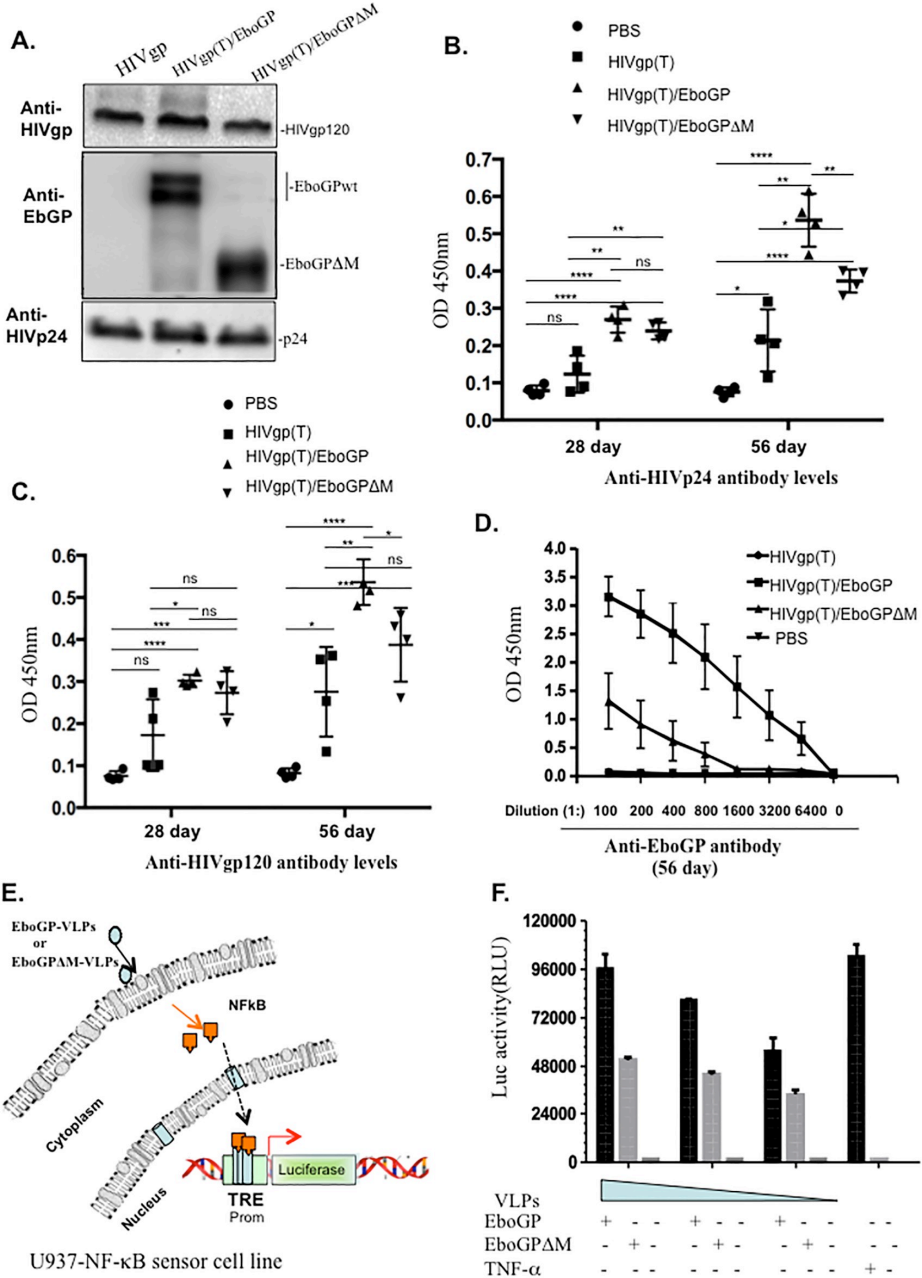
The wild type EboGP-pseudotyped VLPs induced more robust anti-HIV antibody responses than EboGPΔM-pseudotyped VLPs. A) The presence of EboGP, EboGPΔM, and HIVgp in the pseudotyped HIV VLPs were detected by WB. The 293T cells were transfected by CMV-Gag-Pol and HIVgp or EboGPwt/ΔM. **B)** BALB/c mice were injected subcutaneously with 100 ng of HIVgp-VLPs, EboGP/HIVgp-VLPs, or EboGPΔM/HIVgp-VLPs VLPs as indicated. At 21 and 49 days of post-immunization, mice were boosted with same amounts of VLPs. Sera and spleens were collected at 28 and 56 days post-immunization, and levels of anti-HIVgp120 **(B)**, anti-HIVp24 **(C)**, and anti-EboGP **(D**) antibodies were detected via ELISA. **E)** The schematic diagram of NF-κB activity luciferase reporter assay. The U937 cells were transduced by Cignal Lenti particles encoding a luciferase (luc) gene under the control of a minimal (m)CMV promoter (Promo) and tandem repeats of the NF-κB transcriptional response element (TRE). After transduction, cells were incubated with different amounts of EboGP-, or EboGPΔM-pseudotyped HIV VLPs for 12 hrs, and activation of NF-κB signaling was detected by measurement of the luc activity (F). TNF-α treatment of transduced U937 cells were used as positive control. The value (shown in **B, C**) is the individual amount for each mouse of each group. The value (D) shows the average levels of each group from 4 mice with the standard deviations. Statistical significance was determined using unpaired t test, and significant *p* values are represented with asterisks, *≤0.05, **≤0.01, ***≤0.001, ****≤0.0001.

To test whether incorporation of EboGP in HIVgp(T)-VLPs would also improve HIVgp-specific immune responses and to evaluate the consistency of the observations in a different mouse model, we performed similar experiments by injecting BALB/c mice subcutaneously with 100 ng of HIVgp(T)-VLPs, HIVgp/EboGP(T)-VLPs or HIVgp(T)/EboGPΔM-VLPs (Fig 4A) on days 0, 21 and 49. In agreement with the findings in C57BL/6 mice, the results showed that the HIVgp(T)/EboGP-VLPs induced significantly stronger antibody responses against both HIV Gag p24 and gp120 than HIVgp(T)-VLPs in BALB/c mice (Fig 4B and 4C). It is worth noting that strong humoral responses against HIVGagp24 and HIVgp120 were also elicited in mice injected with HIVgp(T)/EboGPΔM-VLPs (Fig 4B and 4C). However, the enhanced level was slightly lower than that of HIVgp(T)/EboGP-VLPs. Consistently, our results revealed that HIVgp(T)/EboGP-VLPs induced significantly stronger antibody responses against EboGP than antibody responses induced by EboGPΔM/HIVGP(T)-VLPs (Fig 4D). These results suggest that EboGP has stronger ability to stimulate immune responses than EboGPΔM, even though EboGPΔM had better targeting ability on MDMs/MDDCs.

To elucidate the possible mechanism(s) responsible for the stronger ability of EboGP to stimulate immune responses, we further investigated whether EboGP-VLPs and EboGPΔM-VLPs could differentially stimulate the NF-κB signaling pathways, since previous studies reported that EboGP is able to activate NF-κB signaling pathways [21,32]. Hence we utilized a NF-κB-Cignal Lenti luciferase report system, in which the lentiviral vector contains a reporter firefly luciferase gene under the control of a minimal (m) CMV promoter and tandem repeats of the NF-κB transcriptional response element (TRE), and generated a U937-NF-κB sensor cell line. Briefly, U937 cells, a human macrophage cell line[33], were transduced with NF-κB-Cignal Lenti particles to generate U937-NF-κB sensor cells, as previously described[34]. Then, the U937-NF-κB sensor cells were treated by equivalent amounts of EboGP- or EboGPΔM-VLPs, and after 48 hrs, cells were lysed and the Luc activity in cell lysates were measured for determination of the activation levels of the NF-κB signaling pathways (Fig 4E). Meanwhile, the U937-NF-κB sensor cells were treated with TNF-α as positive control. The results revealed that EboGP-VLPs induced higher levels of Luc activity than EboGPΔM-VLPs in U937-NF-κB sensor cells (Fig 4F), suggesting that the wild type EboGP has stronger ability to stimulate NF-κB pathways than EboGPΔM. These results are in agreement with a previous report showing that the stimulating effect of EboGP on NF-κB signaling pathway requires the mucin like domain [21].

### Cytokines and chemokines were produced by splenocytes of HIVgp(T)/EboGP-VLP-immunized mice after *in vitro* stimulation with HIV Gag and/or Env peptides

**Fig 5:**
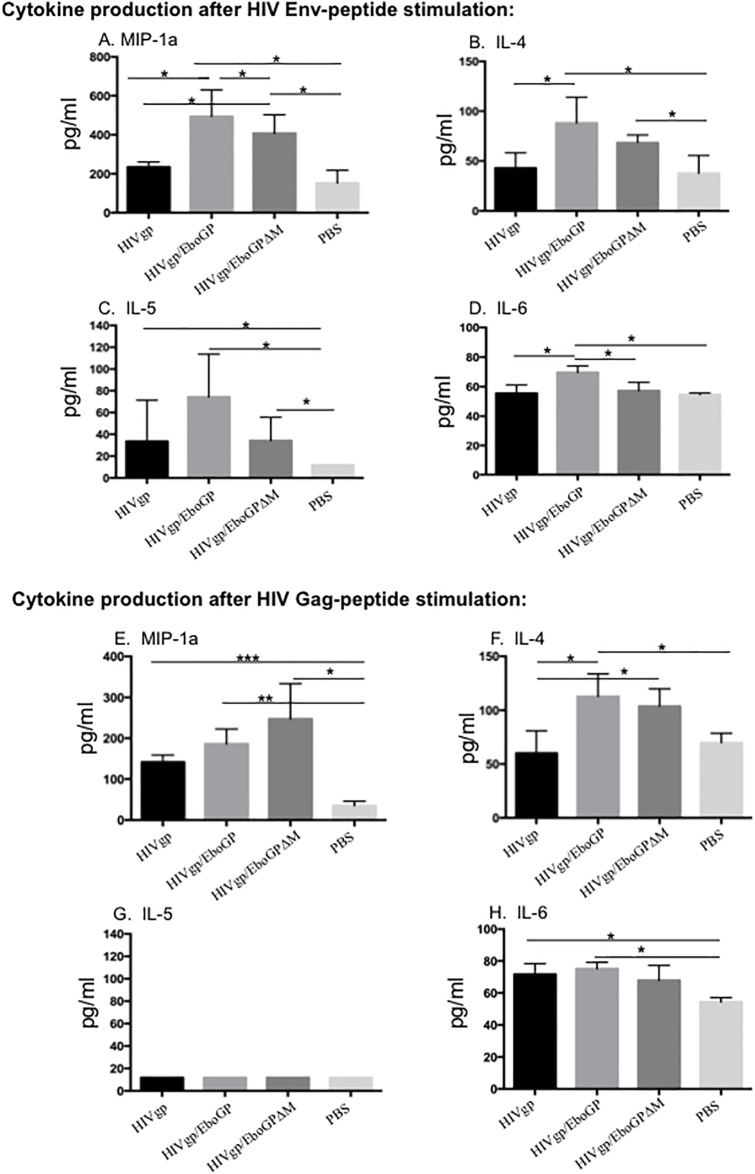
EboGP-HIV VLPs-immunized mice splenocytes produced more profound levels of MIP-1α, and IL-4 after stimulation of HIV Gag and/or Env peptides. Splenocytes isolated from immunized mice were stimulated or non-stimulated with HIV Env (upper panel) or Gag peptides (lower panel). After 72 hrs of stimulation, supernatants were collected and the release of cytokines and chemokines in the supernatants were quantified with a 20-plex mouse cytokine kit and counted in the MAGPIX instrument. Statistical significance between two groups was determined using unpaired t test, and significant *p* values are represented with asterisks, *≤0.05, **≤0.01. No significant (ns) was not shown.

We next evaluated the cell-mediated immune responses against HIV Gag and Env peptides induced upon immunization of mice. Splenocytes isolated from immunized BALB/c mice were stimulated with HIV Gag or Env peptides and the released cytokines and chemokines were quantified using a 20-plex mouse cytokine kit (Invitrogen). Results revealed that all VLPs-immunized mice splenocytes produced significant higher levels of MIP-1α, as compared to PBS-treated mice splenocytes (Fig 5A and 5E). Specifically, after HIV Env peptides stimulation, HIVgp(T)/EboGP-VLPs and HIVgp(T)/EboGPΔM-VLPs immunized mice groups achieved approximately 2-fold higher levels of MIP-1α, than that of HIVgp(T)/VLPs group (Fig 5A). Meanwhile, the results showed that upon Gag-stimulation, splenocytes from HIVgp(T)/EboGP-VLPs and HIVgp(T)/EboGPΔM-VLPs immunized mice produced moderately higher MIP-1α than that of PBS group (Fig 5E). On the other hand, upon both Gag and Env peptides stimulation, increased levels of IL-4 in splenocytes immunized with HIVgp(T)/EboGP-VLPs and/or HIVgp(T)/EboGPΔM-VLPs were also observed, as compared to the HIVgp(T)-VLPs and PBS-immunized mice (Fig 5B and 5F). The level of IL-5 was slightly elevated in HIVgp(T)/EboGP-VLPs-immunized splenocytes after being stimulated by HIV Env peptides (Fig 5C), while no difference in IL-5 levels were observed between all groups after being stimulated by HIV Gag peptides (Fig 5G). Also, a significant increased level of IL-6 was observed in splenocytes from HIVgp(T)/EboGP-VLPs immunized mice when stimulated by HIV Env or Gag peptides (Fig 5D and 5H). It is worth noting that no boosting effect on IFN-γ was observed in all immunized mice after HIV peptide stimulation. The reason remains elusive, but this could be due to the fact that the BALB/c mouse exhibits a characteristic Th-2-like response with dominant AG-specific IL-4 response but no IFN-γ response after stimulation [35]. Overall, these data suggest that the presence of EboGP and/or EboGPΔM in HIV VLPs can enhance the production of MIP-1α, and IL-4, up to HIV Env peptides stimulation.

## Discussion

Although remarkable progress has been made in HIV vaccine development, most HIV vaccines using native Env as an antigen have lacked efficacy conferring protection against HIV acquisition due to the poor T cell and antibody responses [36,37]. To date, the only HIV vaccine trial showing the ability to reduce infection risk was the RV144 trial, which evaluated a regimen comprising a prime with canarypox vectored vaccine (ALVAC) expressing gag/pol/nef and boost with recombinant HIV gp120 [38]. The RV144 vaccine only demonstrated 60% efficacy in infection reduction in the first year of study, subsequently decreasing to 31%. DC-based immunotherapeutic vaccines are a promising approach to address the inadequate immune responses conferred by previously tested HIV vaccines due to their potential to work synergistically with the host immune system to optimize the adaptive response against pathogens.

In this study, we aimed to develop a proof-of-concept vaccine targeting DCs to enhance HIV-specific immune responses by pseudotyping HIV virions with EboGP. Since we wanted to eliminate the capacity of the vaccine to target CD4^+^ T cells as in wild-type HIV infection, we developed one platform in which both EboGP and HIV Env were expressed on the non-replicating virus like particles (VLPs). Thus, the full function of CD4^+^ T cells could be maintained, allowing for the induction of stronger host protective immunity against a multitude of different HIV-1 variants by inducing HIV immunity and eliminating the establishment of chronic infection. We demonstrated that incorporation of EboGP *in trans* by non-replicating HIV VLPs is able to enhance their targeting towards human MDDCs and MDMs, while the deletion of the MLD region of EboGP further enhanced this effect. This finding is consistent with a previous study that showed that the removal of the MLD region greatly enhanced EboGP-mediated lentiviral vector entry into epithelial cells [25]. A potential concern raised by this concept is that DCs have been established as targets for HIV-1 infection, resulting in depletion and dysfunction in their ability to stimulate T-cell proliferation [39]. However, the exact mechanism of DC dysfunction during HIV infection is unknown and some studies suggest that DCs of HIV-infected individuals retain their ability to stimulate allogeneic T-cell responses and their ability produce cytokines upon stimulation with Toll-like receptor 7/8 agonists [40]. Since the HIV VLPs expressing EboGP *in trans* is unable to replicate, it is highly unlikely that these vaccines would cause significant DCs depletion or dysfunction.

In our study, we demonstrated that the presence of EboGP on HIV VLPs increased HIV-specific antibody titers in immunized mice compared with non-pseudotyped HIV VLPs. Although the incorporation of EboGPΔM increased the DC-targeting ability of pseudotyped HIV VLPs compared with EboGP, HIV-specific antibody titers were greater in mice immunized with EboGP-pseudotyped VLPs, suggesting that MLD of EboGP is required for the enhanced immunogenicity of HIV induced by EboGP (Figs 3 and 4). However, even though EboGP promoted increase in anti-HIV antibodies, their ability to neutralize HIV or perform other mechanisms of antibody-mediated protection remains unknown and requires future evaluation.

The HIV-specific cellular response was also enhanced in mice immunized with EboGP/HIV-VLPs, whose splenocytes secreted increased levels of MIP-1α, and IL-4 upon stimulation with HIV Env and/or Gag peptides *in vitro*. Interleukin-4 (IL-4), a marker for the Th2 subset of effector T cells, has many biological roles, including the stimulation of activated B-cell and T-cell proliferation[41]. Therefore, these findings implicated that there is a functional association between EboGP-induced stronger Th2 cytokine production and more efficient anti-HIV humoral responses. However, we did not detect the IFN-γ (a marker for the Th1 response) in the assay. This may be because of the usage of BALB/c mice in this study, which is a typical Th2-dominated response profile in response to IRBP *in vitro*, with high levels of IL-4, no detectable IFN-γ response *in vivo* [35]. Further studies will be required to investigate whether the presence of EboGP could enhance the broad CTL responses, which is also involved in reducing viral loads, thereby contributing to the elimination of productively infected cells. An interesting finding is a significant elevated MIP-1α (CCL3) production in EboGP-pseudotyped HIV VLPs immunized splenocytes. Not only is MIP-1α a potent chemo-attractant for Th1 cells, but it is also the natural ligand of CCR5 that is able to interfere with HIV replication [42]. Previous studies have reported that in some elite controllers, the increased production of MIP-1α and/or MIP-1β at the protein level may be sufficient to confer the cellular resistance to R5-tropic HIV cellular [43,44]. Studies on HIV vaccines have also indicated that the production of chemokines especially MIP-1α and MIP-1β, correlates with protection against HIV [45]. Hence, our findings raise the possibility that a significant increase in production of MIP-1α mediated by action of EboGP-pseudotyped HIV VLPs during vaccination may provide an additional mechanism that may potentially drive cell-mediate protection against R5-tropic virus during the initial stage of HIV infection *in vivo*.

The enhanced HIV-specific immune responses elicited by the EboGP-pseudotyped HIV VLPs suggest its potential to enhance HIV-specific immunity as a novel DC-based vaccine strategy. Our findings that EboGP-pseudotyped HIV VLPs induced increased NF-κB signaling in U937 cells compared with EboGPΔM-pseudotyped HIV VLPs suggests that DC/MDM-targeting and/or the immunostimulatory effects of EboGP could improve the ability of DCs and MDMs to enhance the adaptive immune response. However, a limitation of the study was that NF-κB signaling was only studied in a U937 cell line and not DCs harvested from immunized mice. Further studies are still required to provide direct evidence that the increased HIV-specific adaptive immune responses observed in mice immunized with EboGP-pseudotyped HIV VLPs was a result of enhanced DC-targeting and adjuvant-like effects of EboGP.

Overall, the present study has provided evidence for the first time that the presence of EboGP in HIV VLP enhances its DCs/MDMs targeting ability and can significantly induce robust host immune responses in mouse model. Further optimization of this strategy will lead us to investigate its potential either as therapeutic vaccine (used for *in vivo* administration, and/or by priming DCs *in vitro*) or as a preventive vaccine strategy. Undoubtedly, the outcomes of this study will shed more light on the development of HIV vaccines for controlling and/or curing HIV infection.

## Materials and methods

### HIV-1 constructs and EboGP expressing plasmids

HIV Gag-Pol expressing plasmids (CMVin-Gag/Pol), HIV-1 RT/IN/Env tri-defective proviral plasmid containing a gene encoding for secreted Gaussia luciferase (G-Luc) at the position of *nef* (ΔRI/ΔE/Gluc), the HIV Env glycoprotein expressing plasmids pLET-Lai (X4-trophic) and pLET-JRFL (M-trophic) used in this study were previously described [29]. The codon-optimized (opt) Zaire Ebolavirus glycoprotein (EboGP) expressing plasmid (pCAGGS-optEboGP) was previously described [26]. To construct the mucin-like domain deleted EboGP plasmid (pCAGGS-optEboGPΔM), Two-step PCR was used to amplify optEboGPΔM gene (5’ primer, 5-AATTCGAGCTCGCCACCATG; ΔM primer-3’, 5-TCTAGAGTAGGGCCCTCCTTCCTCGGAACGGATTT; ΔM primer-5’, 5-AGGGCCCTACTCTAGAAACACCATCGCAGGTGTT; 3’ primer, 5-TGCTAGCTCGAGCATGCTCAGA). Then the amplified optEboGPΔM gene was cloned into pCAGGS vector using *Sac*I and *Xho*I sites.

### Cells, antibodies and chemicals

Human embryonic kidney 293T, CD4^+^ C8166 T cells, THP-1 cells or U937 cell line [33] were cultured in DMEM or RPMI-1640 medium supplemented with 10% fetal bovine serum (FBS). To obtain the MDMs and MDDCs, the CD14^+^ monocytes were isolated from hPBMCs by using Easy Sep^™^ Human CD14 positive selection Kit II (STEMCELL Technologies) and were treated with 10 ng/ml of M-CSF or GM-CSF/IL-4 (R&D Systems) for 7 days. The HIV-1 gp120 monoclonal antibody (IG6), and HIV-1 IIIB gp120, p24 recombinant proteins (Cat#11784; Cat#13126) and Consensus Subtype B Env and Gag peptide pools (Cat#12425; Cat#12540) were obtained from the NIH AIDS Research and Reference Reagent Program. Anti-HIVp24 antibody was described previously [29]. Monoclonal antibody (MAb) 42/3.7 against Ebola GP was kindly provided by Dr. A. Takada, Hokkaido University, Japan [46].

### Production and characterization of HIV VLPs

To produce EboGP and/or HIVgp-pseudotyped VLPs, 293T cells were co-transfected with CMV-Gag-Pol expressing plasmid, ΔRI/ΔE/Gluc HIV vector, HIV gp (T/M tropic) and/or EboGP expressing plasmids, as indicated in Fig 1A. At 48 h of post-transfection, supernatants were clarified by centrifugation at 3000 rpm for 15 min, VLP particles were pelleted by ultra-centrifugation, and re-suspended in Endotoxin-free PBS (EMD Millipore Corp). The virus stocks were quantified for HIVGagp24 levels and kept in -80°C for both *in vitro* infection and/or *in vivo* immunization experiments.

To examine the incorporation levels of EboGP and other viral protein in VLP particles, the purified VLPs were lysed, and analyzed by SDS-PAGE and Western blot with MAb 42/3.7, anti-HIVgp120 or anti-HIVp24 antibodies. To test the virus entry ability of different VLP stocks to various cells, equal amounts of HIVgp (T or M tropic)-VLPs or EboGP-VLPs (as adjusted by HIV Gagp24) were used to infect CD4^+^ C8166 T cells, THP-1 cells, MDMs or MDDCs.

### Gaussia luciferase Assay and NF-κB activity luciferase reporter assay

For measuring Gaussia luciferase activity, at various time points after infection as indicated, supernatants from the cell cultures were collected. A 50 μl of GAR-1 reagent (Targeting systems) was added to 20 μl of sample and was mixed well and then measured in the luminometer (Promega, USA) [29].

The VSV-G pseudotyped lentiviral particles (Cignal Lenti vector) expressing a reporter firefly luciferase gene under the control of a minimal (m) CMV promoter and tandem repeats of the NF-κB transcriptional response element (TRE) (Cat# 336851, QIAGEN, Hilden, Germany) were used to transduce U937 cells, a human macrophage cell line [33]. Following transduction, the U937 cells were cultured under puromycin selection to generate a homogenous population of the U937-NF-κB sensor cell line. Then, the U937-NF-κB sensor cells were treated with different EboGP-pseudotyped HIV VLPs or TNF-α for 24 hours, and were lysed and subjected to luciferase assay to monitor NF-κB signaling activity upon various VLP treatments [29].

### Mice immunization experiment

Female C57BL/6 or BALB/c mice, aged 4–6 weeks, were obtained from the Central Animal Care Facility, University of Manitoba (with the animal study protocol approval No. 16-012/1 (AC11159)). The mice (three to four mice per group) were injected subcutaneously with 100 ng (HIVp24) of VLPs in 100 μl endotoxin-free PBS on days 0, 21 and 49 and blood samples were obtained on days 28 and 56. Blood was allowed to clot for 1–2 hrs at room temperature and was centrifuged at 8,600 x g for 3 min. The resulting sera were stored at -20°C until further analysis.

### Anti-HIV and anti-EboGP antibody measurements by Enzyme-linked Immunosorbent Assay (ELISA)

To determine HIV Env and Gag specific antibodies in sera, ELISA plates (NUNC Maxisorp, Thermo scientific) were coated with 100 μl of HIV-1 IIIB gp120 or HIV-1 IIIB p24 recombinant proteins (1μg or 0.5μg/ml) (NIH AIDS Reagent Program, Cat# 11784, Cat#13126) in 0.05M carbonate-bicarbonate buffer of pH 9.6 overnight at 4°C. The plates were washed twice with PBST and blocked with 1% BSA in PBS for 1 hrs at 37°C. 100μl of diluted mouse serum samples (1:100) in PBS were added and incubated for 2 hrs at 37°C. Plates were washed three times and peroxidase-conjugated goat anti-mouse immunoglobulin G (IgG) (GE Healthcare) was added and incubated for 1 h at 37°C. The plates were washed, and 3’,3’,5’,5’ Tetramethylbenzidine (TMB) (Mandel Scientific) was added and incubated for 15 min at room temperature, the reaction was stopped by adding 1N HCL and absorbance was measured at 450nm. Detection of anti-EboGP specific antibodies in the mouse serum with ELISA assay was done as described previously [47]. Briefly, a 96-well EIA plate was coated with purified recombinant EboGP without the transmembrane domain (TM) (IBT Biosciences, Cat# 0501–015), and blocked with 5% skim milk in PBS for 1 hr at 37°C. Then, 30 μl of diluted serum samples were added and incubated for 1 hr at 37°C, followed by adding HRP-conjugated anti-mouse IgG (Southern Biotechnology, Cat # 1030–05) in 2% skim milk, and incubated for 1 hr at 37°C. Then, plates were washed and 50 μl of TMB substrate was added (Thermo Fisher, Cat. 002023) per well and was incubated for 30 min. Plates were read at 650 nm.

### Cytokine detection

Splenocytes from immunized mice were placed into the cell strainer and using the plunger end of the syringe to mash the spleen through the cell strainer into the petri dish to make single-cell suspensions, and cultured in 48-well plates at a density of 2x10^6^/125μl with DMEM containing either HIV-1 Consensus B Gag Peptide pool (3 μg/peptide/ml) or HIV-1 PTE Env peptide pool (3 μg/peptide/ml). Supernatants were harvested following 3 days of culture and stored at -70°C until assay. Cytokine levels (FGF basic IL-1b, IL-10, IL-13, IL-6, IL-12, IL-17, MIP-1a, GM-CSF, MCP-1, IL-5, VEGF, IL-1a, IFN-γ, TNF-a, IL-2, IP-10, MIG, KC and IL-4) were measured in supernatants by using 20-plex mouse cytokine kit (Invitrogen). Briefly, 25 μl of 1x anti-cytokine antibody-coupled beads were added to the 96-well flat bottom plate and the plate was inserted on the magnetic separator for 60 seconds followed by two times wash with wash buffer. Then 50 μl of supernatants and 50μl incubation buffer were added and incubated for 2 hrs at room temperature on an orbital plate shaker. After incubation, the complexes were washed two times using magnetic separator wash procedure followed by incubation with a 1x biotinylated detector antibody for 1 hour. After washing, 1x streptavidin-RPE solution was added into the assay well and incubated for 30 min. Finally, the plate wells were washed three times and the complexes were re-suspended in 150 μl of wash solution and at least 50 beads were counted during the acquisition in the MAGPIX instrument (EPX370-40045-901, Luminex) according to manufacturer’s instructions and xPONENT running protocol setup.

### Statistical analysis

Statistical analysis of levels of antibody/cytokine, including the results of luciferase assays, gp120, p24 ELISA and various cytokine/chemokines were performed using the unpaired t-test (considered significant at *P*≤0.05) by GraphPad Prism 6.01 software.
